# Сase Report of Acute Toxic Imatinib-induced Hepatitis in a Patient with Chronic Myeloid Leukemia, Sulfa Allergy, and Rheumatoid Arthritis

**DOI:** 10.7759/cureus.3136

**Published:** 2018-08-13

**Authors:** Nataliia Lopina, Iryna Dyagil, Dmytro Hamov, Larysa Zhuravlyova, Iryna Dmytrenko, Dmytro Lopin, Kuznetsov Igor

**Affiliations:** 1 Department of Internal Medicine № 3 and Endocrinology, Kharkiv National Medical University, Kharkiv, UKR; 2 Department of Radiation Oncohematology and Stem Cell Transplantation, National Scientific Center of Radiation Medicine of the National Academy of Medical Sciences of Ukraine, Kyiv, UKR; 3 Regional Medical Hematology Center, Cherkasy Regional Oncology Dispensary" of Cherkasy Regional Council, Cherkassy, UKR; 4 Department of Radiation Oncohematology and Stem Cell Transplantation, National Scientific Center of Radiation Medicine of the National Academy of Medical Sciences of Ukraine, Kiev, UKR; 5 Department of Ultrasound Diagnostic, SI Zaitsev V.t. Institute of General and Urgent Surgery of National Academy of Medical Science of Ukraine, Kharkiv, UKR; 6 Kharkiv Medical Academy of Postgraduate Education, KZOZ Kharkiv Regional Hospital/Center for Emergency Medical Care and Disaster Medicine, Kharkov, UKR

**Keywords:** acute toxic hepatitis, acute liver failure, drug induced liver disease, chronic myeloid leukemia, tyrosine kinase inhibitors, imatinib, hormone therapy

## Abstract

The introduction of imatinib has substantially changed the approaches to the therapy of chronic myeloid leukemia. However, this drug can cause hepatic failure and death in rare cases. This report describes a clinical case of acute, toxic imatinib-induced hepatitis in a 56-year-old woman with chronic myeloid leukemia and concomitant sulfa allergy and rheumatoid arthritis. The patient developed acute imatinib-induced hepatitis after three months of treatment with imatinib and three days after increasing the imatinib dosage from 400 mg per day to 600 mg per day, resolving within three months after imatinib discontinuation and prednisolone administration. This confirms the necessity of great caution during imatinib therapy and the monitoring of liver tests. Approximately 25 reports about clinical cases of imatinib-induced hepatitis have been published up to the present.

## Introduction

Chronic myeloid leukemia (CML) is a malignancy of white blood cells. It is a form of leukemia characterized by the increased and unregulated growth of predominantly myeloid cells in the bone marrow and the accumulation of these cells in the blood [1]. Currently, the main treatments for CML are targeted drugs called tyrosine kinase inhibitors (TKIs), such as imatinib, nilotinib, and dasatinib. As a result, long-term survival rates have dramatically improved since 2001. Inhibitors of tyrosine kinases can promote the development of complete molecular remission in patients with CML, which confirms the leading role of the BCR-ABL protein in the development of the disease [1]. TKIs are a class of drugs that block the action of the mutant tyrosine kinase, a product of the chimeric BCR-ABL gene, which has proved oncogenic activity in CML [1]. Imatinib was the first drug in the TKI group allowed for clinical use. Currently, imatinib is the gold standard of first-line therapy for Ph-positive CML patients in the world [1-2]. However, in rare cases, patients develop acute liver failure caused by imatinib therapy [3]. Nowadays, it is an important clinical problem of gastroenterology, hepatology, and hematology due to its association with high-level mortality and liver transplantations that requires further investigations. At present, approximately 25 reports about clinical cases of imatinib-induced hepatitis have been published. However, the predisposing factors of imatinib hepatoxicity have not been identified. Categories of patients who should not be administered imatinib have not been defined yet. Therefore, each case of imatinib hepatoxicity is important for the global medical community. We report a clinical case of acute toxic imatinib-induced hepatitis in a 56-year-old woman with CML and concomitant sulfa allergy and rheumatoid arthritis, which cannot influence the development of imatinib-induced hepatitis. It was hypothesized that the etiology of the patient's liver injury was imatinib-induced hepatitis. The purpose was to discuss the features of imatinib-induced liver injury and current treatment options.

## Case presentation

A 56-year-old woman, a non-smoking teetotaler, was admitted to our hospital in January 2017. Past medical history showed that the patient had rheumatoid arthritis and an allergy to sulfadimezine (at a young age, the syncope reaction occurred several times after taking sulfadimezine). The diagnosis of CML, early chronic phase, intermediate risk group (Sokal score 1.14, Hasford score 1286) was established seven months prior. Imatinib therapy was started three months ago. The patient noted a fever during the first two months after the administration of imatinib therapy, and its cause was not established. She was not taking any drug for rheumatoid arthritis.

The patient's baseline values of serum transaminases prior to imatinib treatment were as follows: alanine transaminase (ALT) - 12 U/l, aspartate transaminase (AST) - 18 U/l, and total bilirubin - 0.33 mg/dL.

After three months of therapy with imatinib, at a dosage of 400 mg per day, an insufficient reduction in the level of BCR/ABL p210 chimeric gene expression in peripheral blood cells was registered (17.241%). This indicated an absence of partial cytogenetic response according to the criteria of LeukemiaNet (2013). The dosage of imatinib was increased from 400 mg to 600 mg per day. This was accompanied by nausea.

In three days after increasing the imatinib dosage, the following changes in biochemical blood analysis were registered: increased levels of ALT - 1155 U/l (normal values less than 31 U/l), AST - 581 U/l (normal values less than 31 U/l), and total bilirubin - 1.99 mg/dL (normal values less than 0.057 to 0.24 mg/dL) (Table 1). Before the imatinib dosage increase, the laboratory values were: ALT – 39.78 U/l, AST – 29.20 U/l, and total bilirubin - 0.14 mg/dL). A complete hematologic response was registered in the clinical blood count at that time: red blood cells – 3.46х10^12^/l (normal values 3.7 to 4.7х10^12^/l), hemoglobin - 116 g/l (normal values 120 to 140 g/l), white blood cells – 3.8х10^9^/l (normal values 3.98 to 10.4 х10^9^/l), neutrophils – 58.9%, lymphocytes – 27.9%, monocytes - 12,0%, eosinophils - 1.0%, and basophils - 0.2%. Gastroenterology and hematology consults were obtained. Chemotherapy was suspended; hepatoprotector (ademetionine 800 mg QD intravenously) and detoxication therapy (rheosorbilact, Ringer's solution) were prescribed. After two weeks of treatment, cholestasis and jaundice appeared, cytolytic syndrome persisted, and, in addition, international normalized ratio (INR) increased up to 1.9. Despite the discontinuation of imatinib, acute hepatitis transformed from the hepatocellular type to the mixed type (ALT - 692.2 U/l, AST - 978.6 U/l, total bilirubin - 10.72 mg/dL).

Screening tests for viral hepatitis markers (HBsAg, anti-HCV antibodies) were negative. A more in-depth examination for markers of viral hepatitis was assigned. The serological markers of hepatitis C virus infection by the enzyme-linked immunosorbent assay (ELISA) - ELISA -anti-HCV-core, ELISA-anti-HCV-NS3, ELISA-anti-HCV-NS4, ELISA-anti-HCV-NS5 also were not detected, as well as serological markers of hepatitis A virus infection – anti-НАV-IgM were not detected. The qualitative HCV RNA and HBV DNA tests were negative. In addition, the markers of cytomegalovirus infection, herpes viruses, and the Epstein-Barr virus were negative. Thus, acute viral hepatitis and the reactivation of chronic hepatitis were excluded. Copper, iron saturation of plasma transferrin, and ceruloplasmin levels were normal.

There were negative markers of autoimmune hepatitis - antinuclear antibodies (ANA), antibodies to smooth muscles (ASMA), and antimitochondrial antibodies (AMA). The patient categorically denied acetaminophen usage or other drugs, including cold medications, categorically denied alcohol.

**Figure 1:**
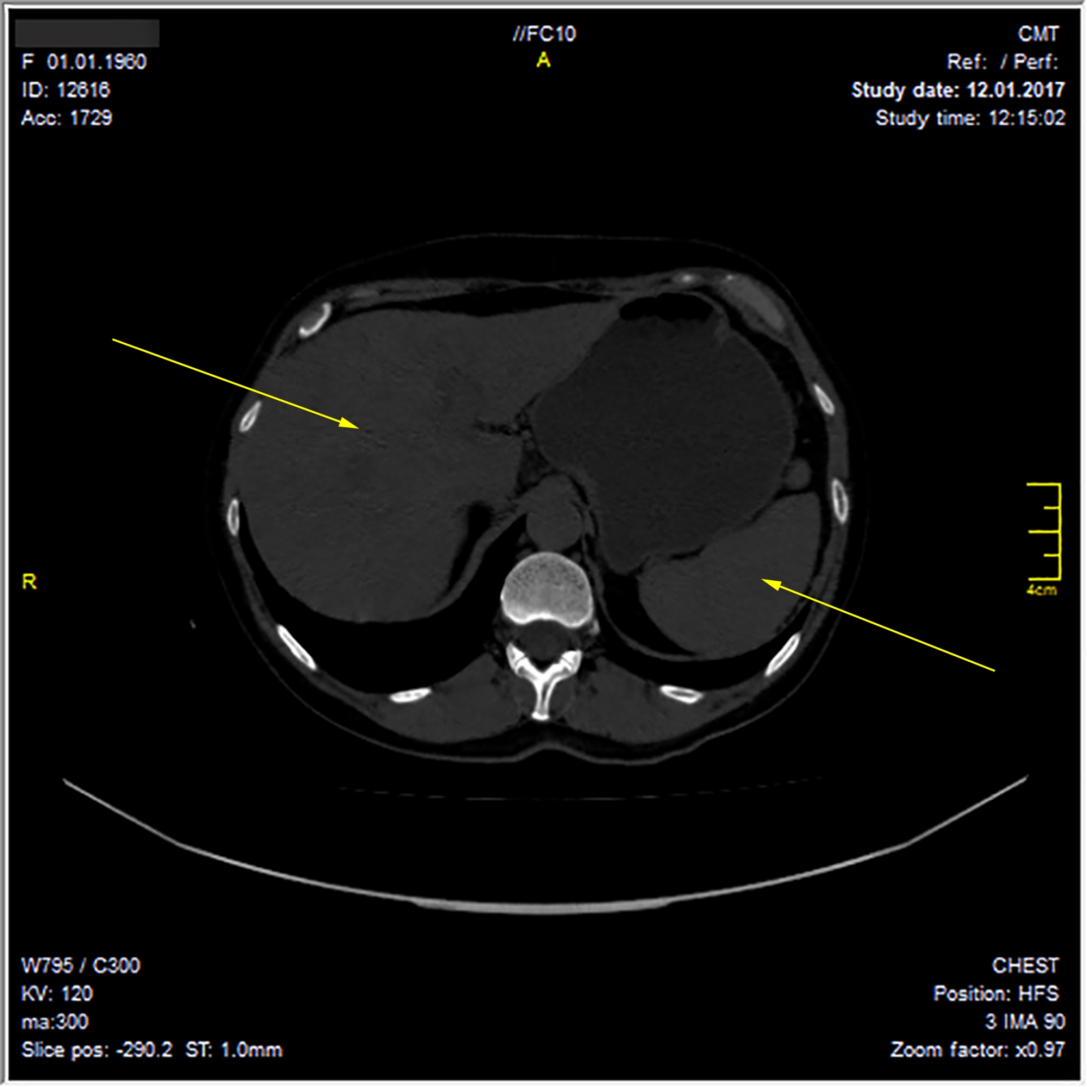
Hepatomegaly (craniocaudal length of the liver was 170 mm), splenomegaly (111x49 mm) according to the computed tomography of the abdominal cavity

The ultrasound examination and computed tomography of the abdominal cavity revealed hepatomegaly (the craniocaudal length of the liver was 170 mm) and hepatic changes similar to hepatitis. Hyperdense liver structures were detected by ultrasonography and periportal edema of the liver parenchyma by computed tomography. The diameter of the common bile duct on ultrasonography was three millimeters. The gallbladder size was 61x27 mm. Intrahepatic bile ducts seen by computed tomography were not dilated. Magnetic resonance cholangiopancreatography was not performed because the common bile duct was not dilated by ultrasonography. The mechanical causes of obstruction were excluded. In addition, splenomegaly was detected (111x49 mm) (Figure 1). A hepatic biopsy was not performed because the patient categorically refused to conduct a liver biopsy.

However, jaundice increased and nausea and vomiting appeared. The laboratory values at that time were: ALT – 538.7 U/l, AST – 563.1 U/l, and total bilirubin – 23.4 mg/dL. It was the highest total bilirubin level after 27 days of imatanib hepatotoxicity beginning. On that date, serum albumin was 34 g/l. The patient was hospitalized for a clarification of diagnosis and a correction of the therapy. Due to the assessment of the grade of hepatotoxicity according to the recommendations of the US National Cancer Institute - NCCN CTC v 5.0 - there was a registered grade III-IV of hepatotoxicity. Due to this, the West-Haven and Conn classification was established as hepatic encephalopathy grade I. According to King’s College Hospital criteria for predicting mortality in acute liver failure in a non-acetaminophen, acute liver injury was confirmed based on three criteria in a reported patient (duration of jaundice to hepatic encephalopathy more than seven days, age more than 40 years, etiology - non-A/non-B hepatitis, drug-induced). The patient had 26 points according to the model for end-stage liver disease (MELD) score.

According to the laboratory and instrumental examinations, the following diagnosis was established:

Acute toxic (drug, imatinib-induced) drug-induced liver disease with cholestatic, cytolytic syndromes, chronic myeloid leukemia, Ph-positive, early chronic phase.

Steroid therapy was initiated, with prednisolone 40 mg per day intravenously. The solutions of ornithine (20 mg/40 ml in 500 ml sodium chloride 0.9 %) and L-arginine 100 ml for detoxication were assigned due to hepatic encephalopathy development. Ringer's solution and intravenous potassium chloride were assigned to reduce the risk of hypokalemia development due to high prednisolone dosage administration. Pantoprazole 40 mg, intravenously, QD, was assigned for the prevention of steroid gastropathy. In addition, a concomitant therapy with ursodeoxycholic acid 750 mg in the evening QD, pancreatic enzymes - pancrelipase 10,000 units three times a day due to high doses, a long duration of pantoprazole administration that reduced the secretion of pancreatic enzymes in the absence of stimulation with hydrochloric acid, and silymarin 140 mg twice daily were prescribed. For the correction of hypoalbuminemia and the prevention of coagulopathy, fresh frozen plasma (once) and albumin (twice) were administered.

Positive clinical and laboratory dynamics was registered during the first week after the beginning of prednisolone therapy: jaundice decreased and bilirubin in the urine disappeared.

**Figure 2:**
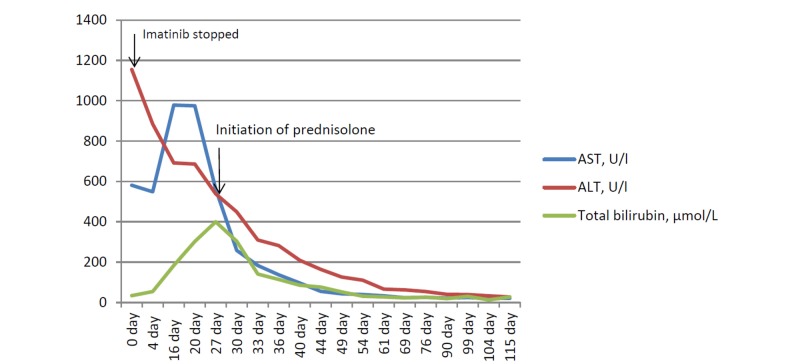
Liver enzyme and cholestatic indicators trend. Hepatic enzyme and cholestatic indicators trend through manifestation imatinib therapy, after discontinuing imatinib therapy, starting steroid therapy AST - aspartate transaminase, ALT - alanine transaminase

After the reduction of cytolysis (AST - 137.4 U/l, ALT - 282.7 U/l and total bilirubin - 6.7 mg/dL) during the second week of the therapy, in addition to prednisolone, a hepatoprotective therapy with ademetionine 800 mg per day intravenously was prescribed, which was then changed to tablets. After 30 days of therapy with prednisolone 40 mg per day intravenously, when the levels of serum transaminases were: AST - 32.9 U/l, ALT - 66.2 U/l, total bilirubin - 1.62 mg/dL, the prednisolone dose was reduced according to the scheme with a switching to the tablet drug form. Due to prednisolone administration, such concomitant prescriptions were made: potassium in tablet form for hypokalemia prophylaxis and pantoprazole in tablets for gastroprotection. A loss of the hematologic response and an appearance of blood leukocytosis 13.2х10^9^/l in the clinical blood count were observed. Due to the loss of the hematologic response, hydroxyurea 500 mg two capsules were prescribed twice per day in addition to the above-mentioned therapy. After a normalization of liver enzymes and cholestasis markers, concomitant therapy was stopped (AST - 22.5 U/l, ALT - 40.3 U/l, total bilirubin - 1.12 mg/dL). Prednisolone was tapered within a month with withdrawal (Figure 2, Table 1). After one month follow-up after discharge from the hospital and prednisolone withdrawal, in a total of three and a half months after the onset of acute toxic hepatitis, acute hepatotoxicity recovered and therapy with nilotinib in a reduced dose of 200 mg twice per day was started because of the loss of the hematologic response (white blood cells 13.2х10^9^/l without hydroxyurea). The laboratory values at that time were: ALT – 33.2 U/l, AST – 20.4 U/l, and total bilirubin – 0.77 mg/dL. A further monitoring of liver functions and clinical blood analysis was performed. It was approved by the hospital's ethics committee in February 2017 for a one-year follow-up period.

**Table 1:** Dynamics of the liver function tests, the conducted measures AST - Aspartate transaminase, ALT - Alanine transaminase, ALP - Alkaline phosphatase, LDH - Lactate dehydrogenase, GGTP - Gamma glutamyltranspeptidase

Day	AST	ALT	Total bilirubin		Unconjugated bilirubin		Conjugated bilirubin		Total protein	ALP	Thymol test	LDH	GGTP	Measures
	U/l	U/l	µmol/L	mg/dL	µmol/L	mg/dL	µmol/L	mg/dL	G/l	U/L	Units	U/L	U/L	
0 day	581	1155	34	1.99	14.5	0.85	19.5	1.14	57.9	183			157	Imatinib therapy was stopped
4 day	548.9	884.4	54.7	3.20	26.3	1.54	28.4	1.66	55.8	212.7	1.1		212.4	Detoxification therapy, hepatoprotectors
16 day	978.6	692.2	183.3	10.72	65.5	3.83	117.8	6.89		210	1.4		253.6	Severe jaundice, adding ursodeoxycholic acid to the therapy
20 day	975.5	686.0	302.3	17.67	101	5.91	201.3	11.76	51.6	173	1.8		195.1	Vomiting, encephalopathy
27 day	563.1	538.7	400.2	23.40	127.9	7.48	272.3	15.92		155.9	1.2	602.4	126.6	Starting administration of prednisolone 40 mg intravenously QD, continued concomitant therapy
30 day (1 month)	258.3	449.1	303.6	17.75	86.2	5.04	217.4	12.71	52.6					Infusion of albumin, fresh frozen plasma
33 day	183.6	310.2	141.6	8.28	38.1	2.23	103.5	6.05	54.4	144.4	2.7	405.6	178.7	
36 day	137.4	282.7	114.6	6.70	34.2	2.00	80.3	4.7	58.6	127.7	3.0		241.1	
40 day	96.9	209.8	85.8	5.02	28.2	1.65	57.6	3.37	55.2	105.6	2.00	353.7	308.4	
44 day	55.7	164.4	76.8	4.49	29.6	1.73	47.2	2.76	57.3	105.9	1.1	405.2	109.2	
49 day	43.8	126.9	53	3.10	19.9	1.16	33.1	1.94	58.1	105.6	2.2	419.8	85.6	
54 day	39.4	111.1	31.7	1.85	18.7	1.10	23	0.75	62					
61 day (2 month)	32.9	66.2	27.7	1.62	12.7	0.74	15.00	0.88	66.6					Initiation of prednisolone dose reduction from 30 day of the teatment according to the scheme. The beginning of hydroxyurea taking two capsules QD according to the account appearance of leukocytosis 13.2х10^9^/l 69 day
23.9	63.0	22.9	1.34	12.8	0.75	10.1	0.59	53.4	105.6	2.2	419.8	85.6	
76 day	26.3	54.2	26.7	1.56	17.4	1.02	9.3	0.54	66.6	95.8	1.8	476.9	58.5	
90 day (3 month)	22.5	40.3	19.2	1.12	13.1	0.77	6.1	0.35	65.7	105.1	1.3	389.0	38.8	A concomitant therapy was stopped, prednisolone stopped
99 day	25	39.3	30.5	1.78	22.0	1.29	8.5	0.49	58	107.4	1.1	361.2	55.7	
104 day	20.4	33.2	13.2	0.77	10.9	0.64	2.3	0.13	67	109.00	1.4	367.2	52.4	Initiation of second-line therapy - nilotinib 200 mg two times daily
115 day	21.2	27.8	28.1	1.64	21.6	1.26	6.5	0.38	67	110.5		403.2	50.1	

## Discussion

An increase in serum aminotransferase levels is typical for 6%-12% of patients with imatinib therapy, but ALT levels more than five times the upper limit of normal (ULN) are registered only in 2%-4% of patients treated for two to six months or more. In addition, liver damage can be accompanied by a slight increase in the serum levels of bilirubin. These abnormalities are usually transient and asymptomatic [1-4]. Currently, more than 25 clinical cases have been described, in which therapy with imatinib was associated with a rare clinically pronounced acute liver damage with jaundice [5-15]. However, the predisposing factors of imatinib hepatoxicity have not been identified. Categories of patients to whom imatinib should not be prescribed have not been defined. The time of the disease onset varied from six days to several years after the initiation of imatinib treatment. However, most frequently, acute liver damage developed within a period of two to six months [5-15]. Rare cases of clinically significant acute liver damage that led to fatal outcomes were reported [7,12]. Thus, therapy with imatinib can be associated with three forms of acute liver damage [16]:

•Transient and usually asymptomatic increase in serum levels of liver aminotransferase during treatment

•Clinically significant acute liver damage with the development of acute hepatitis, acute hepatic failure

•Reactivation of chronic hepatitis B

Hepatotoxicity is usually eliminated by reducing the dose of imatinib or treatment interruption. Nevertheless, the discontinuation of imatinib therapy due to the severe hepatotoxicity development was required in less than one percent of patients [1-4]. Several deaths from liver failure have been reported in patients treated from CML, polycythemia, and in cases with the concomitant use of acetaminophen. In the presented clinical case, a delayed cholestasic reaction that can worsen the patients’ condition was registered despite imatinib discontinuation. An unfavorable outcome may be predicted and the necessity of hormone therapy is confirmed, which has never been discussed earlier.

Great caution is recommended when using imatinib and medicines that are substrates of CYP3A4 and have a narrow range of therapeutic concentrations, as well as medicines containing acetaminophen. Several cases of acute toxic hepatitis, acute hepatic failure development, and a lethal outcome in concomitant imatinib and acetaminophen usage have been reported [17].

An analysis of imatinib hepatotoxicity cases in the future can help to identify predisposing factors, define the group of patients for whom imatinib should be avoided, especially women [3,5-9,12-14,16,18], thereby reducing the number of cases of severe imatinib hepatotoxicity and the high mortality rate associated with it.

In severe hepatotoxicity cases, liver transplantation can be a life-saving treatment approach. The decision should be individualized for each patient based on King’s College Hospital criteria for predicting mortality in acute liver failure [18-20]. Nowadays, many cases of clinical response with a decrease in hepatotoxicity have been described in cases of simultaneous prednisolone administration. In reported clinical cases, liver transplantation was not performed because the West-Haven and Conn classification established hepatic encephalopathy grade I and a rapid positive laboratory trend began after prednisolone administration.

Recurrent hepatotoxicity was associated with re-exposure to imatinib therapy, but according to some authors [11], simultaneous prednisolone therapy can reduce or prevent the recurrence of liver damage and, in some cases, facilitate long-term therapy in spite of the previous history of clinically significant imatinib-induced liver damage. Elevation of the serum transaminases to more than 5 upper limit of normal (ULN) requires a dose reduction or temporary discontinuation of imatinib therapy. In some cases, therapy was resumed, especially with simultaneous prednisolone administration (from 10 mg to 20 mg per day) [10].

Steroid therapy can cause complications such as hypokalemia and acute gastrointestinal bleeding [20]. In this report, the features of the concomitant therapy of such patients are presented, which had not been previously described. The importance of the reported clinical case consists of a detailed description of the therapy for imatinib hepatotoxicity, including concomitant therapy and the necessity of prednisolone prescription in a delayed appearance of the cholestasis syndrome. The limitations of our case were the absence of a liver biopsy, the delayed starting of prednisolone administration, and it being only a single case report, which requires a study of more clinical cases for the evaluation of acute imatinib-induced liver disease for obtaining a better understanding of this problem.

## Conclusions

Imatinib therapy can cause rare, but serious side effects, such as acute toxic hepatitis and acute hepatic failure. Imatinib hepatotoxicity may be related to an interaction of tyrosine kinase inhibitors with a number of other tyrosine kinases besides BCR-ABL. Thus, it is necessary to consider the possibility of hepatotoxicity development if such therapy is administered; to conduct a comprehensive examination, including virologic tests, before the start of treatment; to take into account inter-drug interactions during targeted therapy and concomitant conditions; and carefully monitor liver functions during therapy. In all patients, liver functions should be carefully monitored and the individual risk/benefit ratio should be assessed in each case prior to the initiation of such therapy.
